# HI from the Sky: Estimating harvest index from UAVs combined with machine learning

**DOI:** 10.1093/plphys/kiad611

**Published:** 2023-11-14

**Authors:** Alexandra J Burgess

**Affiliations:** Agriculture and Environmental Sciences, School of Biosciences, University of Nottingham, Sutton Bonington Campus, Loughborough LE12 5NA, UK

Crop yields must increase in order to meet food security goals. Estimating crop yield-related traits accurately and rapidly is therefore of utmost importance, with applications in breeding, precision farming, and yield monitoring. Hight-throughput phenotyping (HTPP) refers to the use of non-invasive techniques to measure physiological and agronomical traits of crop plants. Optical remote-sensing techniques are widely used in HTPP. They encompass data captured from areas of the visible (400–700 nm, captured using red green blue sensors), near infrared (700–1350 nm), red edge (680–730 nm), and shortwave infrared (1350–2500 nm) regions of the spectrum (Gamon et al. 2019). This spectral information has numerous applications within crop monitoring direct prediction of a trait of interest such as pigment content or biomass area, or through using spectral indices, otherwise known as vegetative indices, which correlate spectral reflectance with physiological traits such as green area, portrayed by the normalized difference vegetation index (Fu et al. 2020; Tattaris et al. 2016).

One of the most important components determining crop yield is the harvest index (HI), the ratio between harvestable yield and above ground biomass. However, relative to other yield-related traits, HI is poorly understood. This is partly a result of the fact that HI is traditionally measured via destructive field sampling, which is both time consuming and labor intensive. Within many of the staple crops, such as wheat and rice, HI is approaching the theoretical maximum value. However, rapid screening of HI is valuable within breeding programs and for the temporal evaluation of growth status.

As the name suggests, remote sensing via optical or other techniques permits data to be collected from a distance, without the need for physical interaction with a plant. These sensors may be fixed or in motion. For example, remote sensing from satellite data has many uses within agricultural research; however, in some instances, application is limited by low resolution and fixed measurement times. Therefore, an alternative approach for field data collection is through the use of unmanned aerial vehicles (UAVs). UAVs are able to provide high-resolution spatial and temporal data for a relatively low cost. So far, UAV-based data have been used to estimate crop traits, including seedling emergence, plant height, leaf area index, above-ground biomass, and yield (Tsouros et al. 2019).

Following collection, data analysis can be supported by a variety of techniques, including machine learning approaches. Ensemble learning (EL) refers to a subset of machine learning whereby multiple learning algorithms are combined within a single framework to obtain higher predictive performance. EL methods are able to overcome problems associated with small training sets, such as overfitting, because outputs of independent base models are integrated through secondary learning methods (Zhang et al. 2019). This integration can be facilitated by Bayesian model averaging, in which the posterior probability of each of the basic models are taken as weights for the secondary learning step. As such, Bayesian model averaging is able to overcome uncertainty in the modeling process, leading to a higher estimation accuracy and so has been widely applied for many fields (Shu et al. 2022).

**Figure 1.**
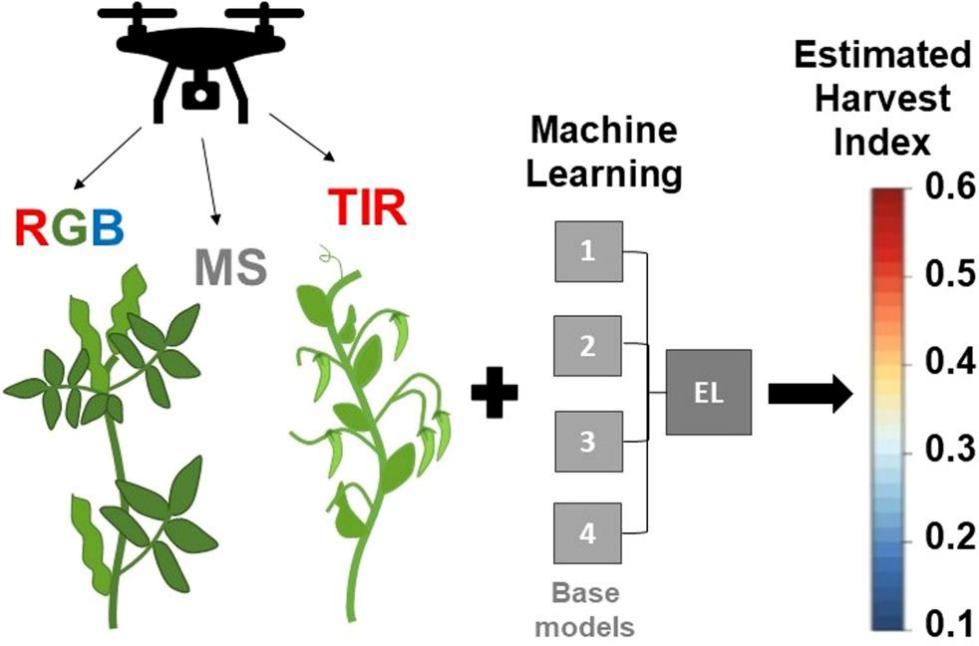
Overview of the remote sensing approach designed by Ji et al. (2023) for the estimation of the harvest index (HI) of field grown fava bean and pea. Optical data were collected using a UAV from red green blue (RGB), multispectral (MS), and thermal infrared (TIR) sensors. Data were analyzed using a variety of machine learning approaches singularly and in combination using ensemble learning (EL) and Bayesian model averaging. The most accurate estimation of HI was obtained using multi-modal data combined with the EL model.

Within this issue of *Plant Physiology*, Ji et al. (2023) used multi-spectral data captured from a UAV combined with machine learning to calculate the HI of pea (*Pisum sativum* L.) and faba bean (*Vicia faba* L.) at 4 different growth stages. Ji et al. (2023) combined data collection from red green blue sensors, multispectral and thermal infrared sensors, as well as 4 different machine learning models and an integrated EL model based on MBA (Fig. 1). Combining multi-modal data provided a better overall estimate of HI compared to any single sensor with improvements in model fitting (i.e. R^2^) of up to 18% and 31% for bean and pea, respectively. Similarly to spectral information, the combined EL model provided the best and most stable estimation of HI, and combining information from all 4 growth stages provided a better estimate compared to any single growth stage.

Together, the results of Ji et al. (2023) indicate the power of combining multi-sensor and multi-model information for the prediction of plant physiological traits. The improved HI prediction power reflects the results of previous studies that demonstrate how multi-sensor information can improve crop trait estimation (Feng et al. 2020; Li et al. 2022). However, species-specific differences were seen in the estimation accuracy of HI, with pea achieving more accurate predictions relative to bean. This improved HI estimation in pea was predicted to be a result of reduced canopy cover and thus reduced saturation of the optical sensors. In some instances, combining information from 2 sensors yielded greater predictive power than 3, indicating possible data redundancy. Furthermore, in some cases, the base machine learning models performed better than the EL approach. Thus the optimal combination of sensors and models is likely to be species, growth stage, and trait specific.

Despite the recent rise in machine learning approaches for plant science disciplines, its application for crop yield prediction is not yet viable for wide-scale use. Nevertheless, although improvements can still be made for the estimation of HI, the study of Ji et al. (2023) presents a further step in the generation of HTPP for monitoring crop performance. Combining low-cost sensor information and machine learning permits robust and accurate measurements of physiological traits, ultimately providing an acceleration in the breeding process. Future improvements will require large datasets encompassing multiple genotypes across a variety of sites and assessment of the redundancy between multi-sensor data.
